# Neuroimaging in Lewy body dementia

**DOI:** 10.1007/s00415-018-8892-x

**Published:** 2018-05-14

**Authors:** Tayyabah Yousaf, George Dervenoulas, Polytimi-Eleni Valkimadi, Marios Politis

**Affiliations:** Neurodegeneration Imaging Group (NIG), Department of Basic and Clinical Neuroscience, Maurice Wohl Clinical Neuroscience Institute, Institute of Psychiatry, Psychology and Neuroscience (IoPPN), King’s College London, 125 Coldharbour Lane, Camberwell, London, SE5 9NU UK

**Keywords:** Lewy body dementia, Neuroimaging, MRI, Molecular imaging, PET, SPECT

## Abstract

Lewy body dementia (DLB) is a common form of cognitive impairment, accounting for 30% of dementia cases in ages over 65 years. Early diagnosis of DLB has been challenging; particularly in the context of differentiation with Parkinson’s disease dementia and other forms of dementias, such as Alzheimer’s disease and rapidly progressive dementias. Current practice involves the use of [^123^I]FP-CIT-SPECT, [^18^F]FDG PET and [^123^I]MIBG molecular imaging to support diagnostic procedures. Structural imaging techniques have an essential role for excluding structural causes, which could lead to a DLB-like phenotype, as well as aiding differential diagnosis through illustrating disease-specific patterns of atrophy. Novel PET molecular imaging modalities, such as amyloid and tau imaging, may provide further insights into DLB pathophysiology and may aid in early diagnosis. A multimodal approach, through combining various established techniques and possibly using novel radioligands, might further aid towards an in-depth understanding of this highly disabling disease. In this review, we will provide an overview of neuroimaging applications in patients with DLB.

## Introduction

Dementia with Lewy bodies (DLB) is a common form of cognitive impairment, accounting for substantial clinical deterioration and a significant burden in patients and caregivers [1]. The classic presentation of DLB encompasses tandem features of fluctuating cognitive decline, parkinsonism and visual hallucinations [1]. Conjointly with Parkinson’s disease dementia (PDD), they comprise a spectrum of neurodegenerative dementias that share the common hallmark of α-synuclein pathology [2]. Thus, the term Lewy body disease is currently used to describe neurodegenerative conditions with similar clinical phenotype (dementia combined with parkinsonism) and underlying pathophysiology [3]. Aggregation of α-synuclein (SNCA) in Lewy bodies and neurites often coexists with amyloid-β plaques and tau neurofibrillary tangles [4]. An integrated approach in these conditions that have a consecutive clinical outcome is ideal for elucidating underlying mechanisms and therefore improving diagnostic tools and therapeutic interventions.

Diagnostic criteria for DLB harbor an acceptable sensitivity [1]. However, specificity and diagnostic accuracy in the clinical setting remain as challenges to be further addressed. In the clinical setting, DLB is often misdiagnosed [5]. Consequently, patients are prone to non-beneficial or even harmful treatment options and incomplete disease management [6]. Clinically relevant biomarkers could potentially contribute to an enhanced diagnostic accuracy [7]. Detecting lower levels of α-synuclein in the CSF of patients with suspected DLB has been proven of potential utility, especially in discriminating from Alzheimer’s disease (AD) [8]. Alas, reliability of CSF or serum biomarkers to serve as positive diagnostic tools is not yet consistent.

In respect of the former and considering that DLB diagnosis relies predominately on clinical features, neuroimaging biomarkers could aid towards an increased diagnostic certainty [9]. Besides excluding secondary causes of dementia using structural imaging, neuroimaging modalities can also be implemented in aiding differential diagnosis and investigating underlying pathophysiological mechanisms (Table 1). However, the application of advanced techniques in the clinical setting requires additional validation. In this review, we will provide an overview of neuroimaging modalities currently used to assess patients with DLB.

**Table 1:** Neuroimaging modalities assessing DLB in the clinical setting

Imaging modality	Application	Main findings
FP-CIT SPECT	Differentiating from AD and HC	Decreased dopamine transporter uptake in the basal ganglia
FDG-PET	Supportive of diagnosis	Reduced glucose metabolism in occipital lobes
CT	Excluding secondary causes of dementia	Intact brain structure
MRI	Differentiating from AD	Relative preservation of medial temporal lobe structures
MIBG	Differentiating from AD	Low uptake

## Diagnosis of DLB

Diagnosis of DLB continues to heavily rely on clinical manifestations of the disease, as structural neuroimaging lacks definitive characteristics with significant diagnostic value [10]. In DLB, cognitive decline either antedates or occurs simultaneously with parkinsonism, whereas in PDD it follows the constellation of parkinsonism. Key characteristics of DLB, which are less common in PDD, include fluctuating cognition and sensitivity to neuroleptics [6]. Supportive features in DLB diagnosis includes relatively preserved medial temporal lobe structures as seen on CT or MRI [10]. Though this feature is commonly present, it has not been proven to have adequate diagnostic specificity. Current diagnostic criteria have included the use of [^123^I]FP-CIT-SPECT, [^18^F]FDG PET and [^123^I]MIBG as supportive or indicative diagnostic features. Recently, besides imaging biomarkers, other clinical measures (polysomnography, electroencephalography) have been incorporated in the diagnostic criteria [9].

## Structural imaging

Structural brain changes can be visualized and assessed using MRI and CT, providing a measure of cerebral atrophy, as well as white matter integrity in DLB. Structural neuroimaging is often utilized in the clinical setting for differential diagnosis of various types of dementia [11]. These imaging techniques are primarily used to detect cerebrovascular diseases and space-occupying lesions such as brain tumor or hematoma [12, 13]. Though CT is most often used clinically due to its relatively low cost and widespread availability, MRI offers superior contrast, as well as specific tissue characterisation (Table 2). An array of analyses have been developed and performed, including whole brain analyses (voxel-based morphometry, cortical thickness), region of interest (ROI) analyses and visual inspection, to compare regional structural changes in patients with DLB to those with Alzheimer’s disease, Parkinson’s disease dementia and healthy controls.

**Table 2:** MR imaging studies in DLB

Study	MR measurement	Assessment	Groups	Findings in DLB patients
Ballard et al. [59]	Visual inspection of white matter lesions on T2-weighted MR images	Hyperintensities	17 DLB, 13 AD	Blood pressure drop > 30 mm Hg was associated with the severity of hyperintensities in the deep white matter and basal ganglia
Barber et al. [55]	Cortical thickness	Atrophy	27 DLB, 25 AD, 24 VaD, 26 HC	DLB patients had larger temporal lobe, amygdala and hippocampal volumes compared to AD, with no volumetric difference compared to VaD. Compared to HC, DLB exhibited relative preservation of whole brain volume
Barber et al. [55]	Ventricular volumes and white matter lesions	White matter hyperintensities	27 DLB, 25 AD	Periventricular hyperintensities correlated with increasing age and ventricular dilation in all subjects. Deep matter hyperintensities were associated with hypertension history
Ballmaier et al. [19]	Cortical thickness	Atrophy	16 DLB, 29 AD, 38 HC	DLB exhibited significantly less gray matter deficits bilaterally in the temporal lobe compared to AD
Burton et al. [14]	Voxel-based morphometry	Atrophy	25 DLB, 30 AD, 25 HC	Loss of gray matter volume bilaterally in the temporal and frontal lobes and insular cortex in patients with DLB compared to HC. Preservation of the medial temporal lobe, hippocampus and amygdala in DLB compared to AD
Burton et al. [30]	Voxel-based morphometry	Atrophy	17 DLB, 26 PDD, 31 PD, 28 AD, 36 HC	PDD exhibited bilateral loss of gray matter in the occipital lobe compared to PD. AD exhibited temporal lobe atrophy, including parahippocampal gyrus and hippocampus, compared to PDD. No differences found between PDD and DLB
Burton et al. [56]	Automated technique using FLAIR image	White matter volume	14 DLB, 13 PDD, 23 AD, 33 HC	AD exhibited increased white matter volume compared to HC. No differences were found in white matter volume in DLB compared to HC, AD or PDD
Sauer et al. [40]	Task-based fMRI	BOLD signal	9 DLB, 10 AD, 13 HC	DLB had increased activation in the superior temporal sulcus compared to AD during the motion task
Beyer et al. [31]	Voxel-based morphometry	Atrophy	18 DLB, 15 PDD, 21 AD, 20 HC	DLB exhibited increased cortical atrophy within the temporal, parietal and occipital lobes compared to PDD. AD exhibited greater atrophy within the temporal and frontal lobe compared to DLB
Whitwell et al. [25]	Voxel-based morphometry & ROI-based analysis	Atrophy	72 DLB, 72 AD, 72 HC	DLB exhibited preservation of the medial temporal lobe, inferior temporal regions as well as hippocampus and temporoparietal cortex compared to AD. Both AD and DLB demonstrated a loss of gray matter in the substantia innominata, though DLB exhibited a greater loss within the midbrain compared to AD
Sanchez-Castaneda et al. [33]	Voxel-based morphometry	Atrophy	12 DLB, 16 PDD, 16 HC	DLB demonstrated increased gray matter atrophy in the right inferior frontal lobe, the right superior frontal gyrus and the right premotor area compared to PDD. Within the DLB group, reduced anterior cingulate and prefrontal volume correlated with worse performance on the Continuous Performance Test, whilst right hippocampal and amygdala volume correlated with Visual Memory Test
Lee et al. [32]	Voxel-based morphometry	Atrophy	18 DLB, 20 PDD	DLB exhibited greater gray matter atrophy in the left occipital, parietal and striatal areas compared to PDD, as well as a greater reduction in white matter density within the occipital and left occipito-parietal areas
Sanchez-Castaneda et al. [34]	Voxel-based morphometry	Atrophy	12 DLB, 15 PDD	DLB with hallucinations exhibited greater loss of gray matter in the right inferior frontal gyrus compared to DLB without hallucinations. Compared to PDD with hallucinations, DLB with hallucinations had a greater reduction of gray matter in the bilateral premotor area, as well as reduced volume in the left precuneus and inferior frontal lobe correlating with visual hallucination
Galvin et al. [35], Chow et al. [21]	Hippocampal radial distance technique	Hippocampal atrophy	16 DLB, 55 AD, 42 HC	DLB demonstrated atrophy predominately within the left CA1 region and subiculum compared to HC
Hayashi et al. [23]	Voxel-based morphometry	Atrophy	60 DLB, 210 AD	DLB exhibited less atrophy in the entorhinal cortex compared to AD
Taylor et al. [41]	Task-based fMRI	BOLD signal	17 DLB, 19 HC	DLB showed reduced functional activation in visual area V5/MT (middle temporal) in response to motion stimuli
Watson et al. [22]	Voxel-based morphometry	Atrophy	35 DLB, 36 AD, 35 HC	Although DLB exhibited gray matter atrophy in occipital, parietal, temporal and subcortical structures when compared to HC, this was to a lesser extent than AD. DLB appeared to have a relative preservation of the medial temporal lobe compared to AD
Watson et al. [22]	Diffusion tensor imaging	White matter integrity	35 DLB, 36 AD, 35 HC	In DLB, reduced FA was identified in parieto-occipital white matter tracts when compared to HC. However, when DLB were compared to AD, reduced FA was observed in the pons and left thalamus. Impaired episodic memory, was associated with increased MD, whilst letter fluency and motor parkinsonism severity were associated with reduced FA
Franciotti et al. [36]	rs-fMRI: ICA	Functional connectivity	18 DLB, 18 AD, 15 HC	DLB had reduced FC in the right hemisphere compared to HC, but not AD, which was found to correlate with severity of fluctuations
Fukui et al. [58]	Visual inspection of T2*-weighted MR images	Cerebral microbleeds quantification	59 DLB, 81 AD	DLB exhibited increased microbleeds in all brain areas, apart from occipital lobe, compared to AD. The number of microbleeds was positively correlated with the severity of white matter lesions in both DLB and AD
Kenny et al. [38]	rs-fMRI: seed-based	Functional connectivity	15 DLB, 16 AD, 16 HC	DLB and AD exhibited increased thalamic and caudate FC compared to HC. DLB patients showed greater putaminal connectivity compared to AD and HC
Lebedev et al. [18]	Cortical thickness	Atrophy	97 DLB, 97 AD	DLB and AD were differentiated with a sensitivity of 82.1% and specificity of 85.7%. Cortical thinning, in DLB, was localized to the middle and posterior cingulate, lateral orbito-frontal and superior temporo-occipital regions
Mak et al. [50]	Cortical thickness and subcortical volumes	Atrophy	13 DLB, 23 AD, 33 HC	AD exhibited greater hippocampal atrophy compared to DLB. In DLB, cortical thinning in the frontal and parietal regions correlated with a decline in global cognition and motor deterioration
Peraza et al. [49]	rs-fMRI	Functional connectivity	18 DLB, 12 PDD, 17 HC	When compared to HC, DLB had greater FC alterations than PDD for seeds situated within the fronto-parietal network. No differences were found when DLB and PDD were compared directly
Watson et al. [17]	Cortical thickness	Atrophy	31 DLB, 30 AD, 33 HC	In DLB, cortical atrophy was less diffuse compared to AD, though cortical change was found to predominately affect posterior structures (inferior parietal, fusiform gyrus and posterior cingulate). In DLB, the average reduction medial temporal lobe cortical thickness was less (6–10%) compared to AD (15–24%)
Delli Pizzi et al. [24]	Cortical thickness and subcortical volumes	Atrophy	19 DLB, 15 AD, 19 HC	The cornu ammonis and subiculum were bilaterally preserved in DLB compared to AD. The perirhinal cortex and parahippocampus were damaged in DLB, but preserved in AD
Watson et al. [53]	Cortical thickness and subcortical volumes	Atrophy	33 DLB, 32 AD, 35 HC	DLB exhibited volumetric loss in the bilateral putamen, left thalamus and total subcortical gray measures compared to controls. AD exhibited a more pronounced loss of gray matter volume in the left pallidum, right thalamus and bilateral amygdala and hippocampus compared to DLB
Shams et al. [27]	SWI analysis	Iron	19 DLB, 20 AD, 20 FTD, 17 MCI, 21 HC	An abnormal swallow tail sign was most common in DLB, with a predictive value only in DLB

*DLB* Dementia Lewy bodies, *AD* Alzheimer’s disease, *HC* healthy controls, *PD* Parkinson’s disease, *PDD* Parkinson’s disease dementia, *FTD* frontotemporal dementia, *MCI* mild cognitive impairment, *VaD* vascular dementia, *rs-fMRI* resting state functional MRI, *FC* functional connectivity, *FA* fractional anisotropy, *MD* mean diffusivity, *BOLD* blood-oxygen dependent, *SWI* susceptibility-weighted imaging

### Comparison between DLB and AD

MRI has been widely used to investigate patterns of gray matter (GM) atrophy. Advances in image processing have enabled automatic extraction of whole brain cortical thickness, which is retrieved from structural MRI. Although DLB has demonstrated some overlap with the cortical atrophy patterns seen in AD, atrophy is generally less diffuse in DLB with moderate preservation of the medial temporal lobe structures [14–16].

Cortical thickness assessment has also been shown to have high precision and sensitivity in identifying morphological changes, which arise from neuropathological changes. This method has, therefore, been employed in several studies as a way to differentiate DLB from AD, PDD and healthy controls. Investigations into cortical thickness alterations in DLB revealed relatively small GM change, primarily affecting the posterior parietal areas, as opposed to the patterns of GM change affecting the temporoparietal association cortices in AD [17]. These findings are in corroboration with the notion that DLB is a result of neuronal synaptic dysfunction, not neuronal loss. Through carrying out a multivariate classification study of cortical thickness, Lebedev and colleagues demonstrated that this method has the ability to differentiate DLB from AD with 82.1% sensitivity and 85.7% specificity [18]. Specifically, AD was characterized by patterns of cortical thinning within the temporal pole, subgenual cingulate regions and the parahippocampus, whereas regional thinning was localized to the superior temporo-occipital and lateral orbito-frontal regions, as well as the middle and posterior cingulate in DLB [18]. The finding of AD exhibiting greater temporal involvement compared to DLB has been a homogenous result across several structural imaging studies [19, 20].

Although investigations into hippocampal atrophy have revealed that DLB patients have less severe atrophy compared to AD patients [21, 22], with the entorhinal cortex, CA1 and subiculum areas of the hippocampus being most affected in AD [23], recently, Delli Pizzi et al. explored the differential contribution of hippocampal subfields and adjacent extrahippocampal structures to the pathophysiology of AD and DLB [24]. They reported that the cornu ammonis and subiculum were preserved in DLB, but the perirhinal cortex and parahippocampus were damaged, highlighting the differential alteration of hippocampal subfields and adjacent extrahippocampal structures in DLB and AD [24].

Studies have also demonstrated greater atrophy in the substantia innominate, with increased dorsal mesopontine GM atrophy distinguishing patients with clinically diagnosed DLB from AD [25]. These findings may suggest a greater cholinergic dysfunction in DLB, perhaps related to the presence of midbrain synuclein pathology.

Using diffusion tensor MRI, Watson et al. revealed that the parieto-occipital white matter tracts were preferentially affected in DLB, though this appears to be an early phenomenon, as AD demonstrated a greater longitudinal increase in mean diffusivity in parietal and temporal regions compared to DLB, with no evidence of longitudinal changes in mean diffusivity or fractional anisotropy in DLB relative to controls [26]. However, DLB was differentiable from AD given that it was associated with reduced fractional anisotropy in the pons and left thalamus, highlighting that, despite similar levels of dementia severity, patterns of DTI changes in DLB and AD varied [26].

A recent study by Shams et al. demonstrated that MRI of the swallow tail sign may have diagnostic potential in DLB [27], given that the largest dopamine-containing cluster within caudal and posterolateral part of the substantia nigra (nigrosome 1) is highly affected in parkinsonian syndromes. More specifically, Shams et al. reported that a hypointense nigrosome 1, as visualized on iron-sensitive susceptibility-weighted imaging (SWI), was more common in DLB compared to AD, frontotemporal dementia and controls. This was in corroboration with Kamagata et al. who reported that measuring nigrosome 1 hypointensity with SWI achieved 90% diagnostic accuracy (93% sensitivity and 87% specificity) in DLB [28].

### Comparison between DLB and PDD

Attempts to compare GM loss between DLB and PDD have revealed a pattern of more pronounced GM loss in DLB compared to PDD, which is in line with the fact that DLB encompasses greater amyloid burden [29]. It is important to note, however, that localisations of GM reductions in DLB relative to PDD vary amongst different studies. For example, Burton et al. were unable to identify distinct cortical atrophy profiles of DLB and PDD [30], but Beyer et al. reported GM reductions in the temporal, parietal and occipital lobes in DLB using a voxel-based morphometry (VBM) approach [31]. Alongside the temporal and parietal atrophy, Lee et al. also reported occipital and striatal GM reductions in DLB [32]. Studies investigating correlation patterns between brain structure and clinical and neuropsychiatric manifestations of the disease, revealed that decreased GM volume of the anterior cingulate, right hippocampus and amygdala were associated with cognitive performance [33], whilst reduced GM volume in the left precuneus and inferior frontal lobe correlated with visual hallucinations in DLB, but not in PDD [34].

## Functional imaging

Active task and resting state functional MRI (fMRI) are the primary tools employed to investigate cerebral function associated to cognitive tasks or during rest, respectively, by measuring changes in blood-oxygen level-dependent (BOLD) signal.

### Comparison between DLB and AD

Although only a few fMRI studies have examined BOLD signal in DLB, differential patterns of functional connectivity in DLB compared to AD have been reported (Table 2). Using the precuneus as the seed region, Galvin et al. reported that DLB patients exhibit increased connectivity in the inferior parietal cortex and putamen, and decreased connectivity in the fronto-parietal operculum, medial prefrontal cortex and the primary visual cortex compared to AD, whilst a reversal of connectivity was observed in the right hippocampus [35]. Independent component analysis (ICA) has demonstrated that DLB display greater connectivity in the default mode network compared to AD [36], which contrasts with previously reported connectivity dysfunctions between anterior and posterior segments of the default mode network in AD, when compared to healthy controls [37]. Furthermore, increased connectivity between the putamen and frontal, temporal and parietal regions has been illustrated by DLB patients in comparison to AD patients, with the authors suggesting that this may be related to the prominent parkinsonian features in DLB [38]. Consistent with the moderate preservation of memory function observed in DLB as opposed to AD, hippocampal connectivity has not been shown to differ in DLB compared to healthy controls, though the left hippocampal connectivity was identified to be higher in AD compared to controls, potentially reflecting a compensatory mechanism [38].

A recent study by Schumacher et al. aimed to explore within- and between-network connectivity in a range of resting state networks, being the first to investigate how DLB affects connectivity between these resting state networks [39]. DLB patients displayed more decreases in within-network connectivity compared to controls, primarily in temporal, motor and frontal networks. In contrast, long-range functional connectivity appeared to be intact in DLB, with increased connectivity only identified between a frontal and a temporal network [39]. Only subtle differences were observed when AD and DLB were compared, suggesting a potential overlap in their resting state functional connectivity.

Given the prominent prevalence of visuoperceptual impairments in DLB, a task-based fMRI study employed visual presentations of motion, color and face paradigms to explore the functional integrity of the visual system in DLB. They discovered that DLB patients exhibited greater activation in the superior temporal sulcus compared to AD, specifically during the motion task [40]. However, these findings were not replicated by Taylor et al., who reported that DLB patients did not exhibit any significant differences in functional response to objects, motion stimuli or checkerboard in V1 and V2/V3 compared to controls [41], proposing that function in the lower visual areas is relatively preserved. Interestingly, however, ROI analysis demonstrated that the DLB group had a reduction in V5/MT (middle temporal) activation when responding to motion stimuli [41]. Taken together, these results imply that, in DLB, functional abnormalities affect the visual association areas, as opposed to the primary visual cortex, though it is difficult to decipher whether deviations at higher levels of the visual system contribute to the hallmark visuoperceptual impairments and visual hallucinations seen in DLB.

### Comparison between DLB and PDD

Although studies have demonstrated alterations in functional connectivity in PDD [42–45] and DLB [35, 38, 46–48], these were reported when comparing these disease groups against healthy controls. One study has, however, compared DLB and PDD directly with the aim of identifying disease-specific functional connectivity patterns (Table 2). Peraza et al. reported that, for seeds situated within the fronto-parietal network, DLB patients exhibited greater alterations in functional connectivity than PDD when compared to healthy controls, predominately at the precentral and postcentral gyri, cerebellar, occipital and temporal regions, whilst in PDD, changes in functional connectivity were limited to the frontal cortices and precuneal [49]. Interestingly, although the supplementary motor area seed revealed similar regional functional connectivity alterations in the pre- and postcentral gyri, cerebellar, temporal, precuneal and occipital regions, these alterations were more apparent in PDD than in DLB, potentially reflecting the prominent parkinsonism and motor dysfunction in PDD compared to DLB [49]. However, Peraza et al. reported that no significant differences were found when DLB and PDD groups were compared to each other. Taken together, these results suggest that there are subtle functional differences between both diseases, which may be driven by their distinct pathological trajectories, thus potentially reflecting the chronological manifestation of cardinal symptoms in the Lewy body dementias.

## Cortical and subcortical involvement in DLB

Serial MRI is an appealing biomarker of neurodegeneration and can assist in monitoring disease progression. Although longitudinal cerebral atrophy rates in AD are well-established and employed as outcome measures in clinical trials of potential disease-modifying agents, the atrophy rate in DLB has been reported to be analogous to or marginally greater than healthy controls [50]. While longitudinal studies of DLB are challenging given the higher mortality rates compared to AD [51], further investigations into DLB patients with a more rapidly progressive disease would be valuable in elucidating the neurobiological underpinnings of disease heterogeneity in DLB.

Evidence of subcortical involvement in DLB has revealed the vulnerability of the thalamus, striatum and brainstem to Lewy-related pathology. Studies have demonstrated that thalamic diffusion and perfusion deficits are associated with DLB [52], and striatal volumetric loss appears to be more affected in DLB than AD [53], with prominent nigrostriatal dysfunction [54]. Significant reductions in brainstem volume in DLB have also been reported [53], with Seidel et al. showing marked to severe neuronal loss in the ventral tegmental, pedunculopontine nucleus and locus coeruleus regions in DLB [54].

## Cerebrovascular pathology

Although cerebrovascular pathology is common in older people, the contribution of vascular lesions to dementia remains to be elucidated. White matter damage can be visualized as focal punctate areas of high intensity signal using T2-weighted MRI. White matter hyperintensities (WMH) burden has been reported to be similar in DLB and AD [55], with DLB displaying no longitudinal change overtime relative to controls and baseline WMH burden predicting progression [56]. Interestingly, a study carried out by De Reuck et al. using a 7-Tesla scanner revealed that DLB patients had more cerebral microinfarcts compared to controls, with a higher abundance of the smallest lesions than vascular dementia and AD [57].

Cerebral microbleeds can be visualized using gradient-echo T2*-weighted MRI. A higher number of microbleeds has been reported in DLB than in AD, aside from the occipital lobes in one study [58]. DLB subjects with microbleeds have less abnormal MIBG scans, indicating that there is an inverse association between vascular lesions and Lewy body pathology [58]. Although Ballard et al. revealed that WMH in the basal ganglia and deep white matter appear to be associated with orthostatic hypotension in DLB [59], more work is required to evaluate the influence of vascular pathologies to the dementia syndrome, clinical features of DLB and its rate of progression.

## Sensitivity and specificity of structural imaging modalities in pathologically proven DLB cases

Though scarce, studies have investigated the diagnostic accuracy of MRI for discriminating DLB from other dementias in autopsy-confirmed cases (Table 4). Both longitudinal and cross-sectional studies have illustrated that DLB is associated with less conspicuous global atrophy, compared to AD, with relative preservation of the medial temporal lobe [22]. Burton et al. aimed to determine the clinical relevance of visually rating the medial temporal lobe on MRI, and whether this technique could serve as an accurate diagnostic tool to distinguish AD from DLB and vascular cognitive impairment (VCI) [16]. In pathologically confirmed cases, medial temporal lobe atrophy served as a highly accurate diagnostic marker, with a sensitivity of 91% and specificity of 94%, in AD compared with DLB and VCI [16]. Medial temporal lobe atrophy scores did not differ between DLB and VCI. These results highlight that medial temporal lobe atrophy on MRI has robust discriminatory power for distinguishing AD from DLB. Furthermore, Burton et al. reported that medial temporal lobe atrophy is pathologically more strongly associated with neurofibrillary tangles and β-amyloid plaques, as opposed to Lewy body-like neuronal inclusions. These results are suggestive of gray matter atrophy, in DLB, arising as a result of concomitant AD-specific pathology. On the contrary, another postmortem MRI study assessing medial temporal lobe atrophy reported that this technique lacked discriminative potential, possessing an inability to exclude DLB diagnosis, particularly amongst patients who were over 85 years of age [60]. Although a strong relationship was found between medial temporal lobe atrophy and Alzheimer’s disease pathology, the sensitivity and specificity were 63 and 69%, respectively, for AD. Medial temporal lobe atrophy was also identified in subjects presenting with alternative primary hippocampal pathology, including Lewy-related pathology, highlighting the lack of specificity for AD-type pathology [60].

Recently, Harper et al. employed structural MRI and 184 post-mortem confirmed dementia cases to evaluate the reliability of six visual rating scales, including the medial temporal lobe atrophy scale, posterior atrophy scale, the anterior temporal scale, orbito-frontal, fronto-insula and anterior cingulate [61]. Using automated classification based on all six visual rating scales, the authors were able to distinguish pathological groups with an accuracy ranging from 86–97% from healthy controls, with DLB being distinguishable with sensitivity of 64% and specificity of 92%, leading to a balanced accuracy of 78% (Table 4). DLB was also differentiated from AD with a sensitivity of 64%, specificity of 82% and balanced accuracy of 73%, as well as from frontotemporal lobar degeneration (FTLD) with a sensitivity of 93%, specificity of 89% and balanced accuracy of 91%. The low sensitivity in distinguishing DLB from controls or AD ultimately emphasizes the elevated number of false negatives attached to DLB diagnosis, which is likely due to the large degree of overlap which exists between DLB and AD, as demonstrated by the fact that ~ 50% of DLB cases exhibit significant amyloid burden [62]. This was also demonstrated by Nedelska et al., who, in histopathologically confirmed cases, demonstrated that mixed DLB/AD cases exhibited markedly higher rates of brain atrophy, with the topography of changes corroborating with that seen in AD, predominantly affecting temporoparietal cortices, amygdala and hippocampi [63]. However, DLB patients exhibited minimal global atrophy compared to controls, with no region-specific atrophy that enabled distinguishability from controls [63]. The issue of false negative diagnoses has critical treatment implications, as failure to properly diagnose DLB clinically will likely result in limited use of existing symptomatic treatments, as well as exposure to non-beneficial or even harmful treatment options.

## Molecular imaging

Molecular imaging has provided further insights into the pathophysiology of a complex disease such as DLB. Modalities such as single photon emission tomography (SPECT) and positron emission imaging (PET) are valuable methods of assessing neurobiology in vivo. Radionucleosides tracing neurotransmitters, synaptic pathology and misfolded protein aggregation provide elusive tools in investigating underlying disease mechanisms (Table 3).

**Table 3:** In vivo PET imaging studies in DLB

Study	Target	Radioligand	Groups	Findings in DLB patients
Rowe et al. [100]	Amyloid	[^11^C]PIB	10 DLB, 17 AD, 6 FTD, 9 MCI, 27 HC	Cortical PIB binding was markedly elevated in every AD subject. In DLB, binding was lower, more variable and correlated inversely with the interval from onset of cognitive impairment to diagnosis
Edison et al. [67]	Amyloid	[^11^C]PIB	13 DLB, 12 PDD, 10 PD, 41 HC	Increase in mean brain uptake
Gomperts et al. [94]	Amyloid	[^11^C]PIB	8 DLB, 7 PDD, 11 PD, 15 AD, 37 HC	Global cortical amyloid burden was higher in DLB than PDD/PD and comparable to AD
Maetzler et al. [98]	Amyloid	[^11^C]PIB	9 DLB, 12 PDD, 14 PD	[^11^C]PIB binding was decreased in all PD (non-demented) [^11^C]PIB binding was increased in 4 PDD and 4 DLB Increased [^11^C]PIB binding was associated with MMSE scores, ApoE4, age, and onset of parkinsonism and dementia
Foster et al. (2010)	Amyloid	[^11^C]PIB	6 DLB, 9 HC, 8 PD-noCI, 9 PD-MCI, 15 PDD	Regional [^11^C]PIB binding potentials were not significantly different across participant groups. Elevated binding was associated with worse global cognitive impairment in participants with Lewy body disorders but was not associated with any other clinical or neuropsychological features
Shimada et al. (2012)	Amyloid	[^11^C]PIB	8 DLB, 7 PDD, 13 AD,17 HC	Amyloid deposition was associated with AD-like atrophy in DLB/PDD patients
Ikonomovic et al. (2012)	Amyloid	[^11^C]PIB	1 Probable DLB and possible AD, 1 Probable AD	[^11^C]PIB was negative in probable DLB, with scarce amyloid plaques identified in less than 2% of the cortical area at autopsy [^11^C]PIB was positive in probable AD with plaques in up to 12% of the cortical area
Gomperts et al. [97]	Amyloid	[^11^C]PIB	18 DLB, 29 PD-noCI, 14 PD-MCI, 12 PDD,85 HC	[^11^C]PIB uptake was higher in DLB than in any of the other groups No differences in [^11^C]PIB uptake across PDD, PD-MCI, PD and HC [^11^C]PIB increased with age and ApoE4 in all patient groups
Sarro et al. [95]	Amyloid	[^11^C]PIB	20 DLB	Higher binding at baseline was predictive of faster cortical, striatal degeneration
Villemagne et al. (2011)	Amyloid	[^18^F]Florbetaben	7 DLB, 20 MCI, 30 AD, 11 FTLD, 5 PD, 4 VaD,32 HC	[^18^F]Florbetaben was positive in 29% of DLB. Cortical binding distribution was similar to AD, however cortical uptake was lower than in AD [^18^F]Florbetaben was negative in all PD
Claassen et al. (2011)	Amyloid, Glucose metabolism	[^11^C]PIB [^18^F]FDG	3 DLB, 3 MSA, 12 HC	All DLB had increased [^11^C]PIB uptake. No [^11^C]-PIB uptake in MSA In DLB, there was correspondence between areas with hypometabolism and high [^11^C]PIB uptake
Iannaccone et al. (2013)	TSPO	[^11^C]PK11195	6 DLB, 11 HC	Increased microglia activation in caudate, putamen, thalamus, substantia nigra, cortex and cerebellum
Albin et al. (1996)	Glucose metabolism	[^18^F]FDG	6 DLB	FDG-PET revealed diffuse cerebral hypometabolism in both pure DLBD and DLBD-AD, with marked declines in association cortices and relative sparing of subcortical structures and primary somatomotor cortex. These subjects also had hypometabolism in the occipital association cortex and primary visual cortex
Imamura et al. (1997)	Glucose metabolism	[^18^F]FDG	19 DLB, 19 AD	In DLB patients, when compared with AD patients, significant glucose hypometabolism was distributed in the temporo-parieto-occipital association cortices and the cerebellar hemispheres. In contrast, glucose metabolism in the medial temporal and cingulate was significantly lower in AD patients
Ishi et al. (1998)	Glucose metabolism	[^18^F]FDG	12 DLB, 12 AD, 12 HC	Glucose metabolism was significantly lower in patients with DLB than HCs in most parts of the brain, except the sensorimotor cortices, basal ganglia, thalamus, and pons. Between the DLB and AD groups, there were significant regional differences in glucose metabolism in the medial and lateral occipital lobes. Occipital glucose metabolism was a useful measure for the differential diagnosis of DLB from AD. The sensitivity and the specificity were 92% when using the minimal value of the normalized occipital metabolism in the HC group as the cutoff point
Higuchi et al. [119]	Glucose metabolism	[^18^F]FDG	6 probable DLB, 1 DLB, 11 probable AD, 10 HC	Among widespread cortical regions, glucose hypometabolism was evident in the DLB group. The metabolic reduction was most pronounced in the visual association cortex compared to that in the AD group. Using a metabolic ratio of 0.92 in the visual association cortex as a cutoff, DLB could be distinguished from AD with a sensitivity of 86% and a specificity of 91%
Liu et al. [71]	Glucose metabolism, amyloid	[^18^F]FDG, [^11^C]PIB	37 DLB, 5 HC	DLB subjects exhibited cortical amyloid deposition and hypometabolism in the bilateral temporo-parieto-occipital region, insula, precuneus, posterior cingulate, frontal lobe and caudate nuclei
Gomperts et al. [102]	Tau, amyloid	[^18^F]AV1451, [^11^C]PIB	7 DLB, 8 PD-impaired, 9 PD-normal, 29 HC	In patients with DLB, cortical [^18^F]AV1451uptake was highly variable and greater than in the HC, particularly in the inferior temporal gyrus and precuneus. Elevated cortical [^18^F]AV1451 binding was observed in 4 of 17 patients with Lewy body disease with low cortical [^11^C]PIB retention. For DLB and PD-impaired patients, greater [^18^F]AV1451 uptake in the inferior temporal gyrus and precuneus was associated with increased cognitive impairment as measured with the MMSE and the CDR
Shimada et al. (2015)	AChE	[^11^C]MP4A	14 DLB, 25 AD, 18 HC	Mean cortical cholinergic activity in AD patients (8.2%) compared with normal subjects and DLB patients (27.8%) was lower than HC. There was a significant difference in mean cortical cholinergic activity between AD and DLB patients
Gilman et al. (2004)	VMAT2	[^11^C]DTBZ	20 DLB [Six developed parkinsonism at least 1 year before dementia (DLB/PD) and 14 developed dementia before parkinsonism or at about the same time (DLB/AD)], 25 AD, 19 NC	Striatal mean binding potential was decreased by 62%–77% in the DLB/PD group and 45%–67% in the DLB/AD compared to AD and control. Binding was lower in the DLB/PD group than the DLB/AD. Both DLB groups had an anterior to posterior binding deficit gradient relative to controls, largest in posterior putamen, smaller in anterior putamen, smallest in caudate nucleus. PET with (+)-[^11^C]DTBZ differentiates DLB from AD, and decreased binding in AD may indicate subclinical DLB pathology
Koeppe et al. (2008)	VMAT2	[^11^C]DTBZ	25 DLB, 30 PD, 25 AD, 57 NC	[^11^C]DTBZ distribution volume was decreased significantly in caudate nucleus, anterior putamen, and posterior putamen in DLB and PD compared with AD and NC. The gradient was significantly steeper in PD than DLB. Both PD and DLB showed significantly greater interhemispheric striatal binding asymmetry than NC. PD had greater asymmetry than DLB. Greater reduction of K1 occurred in occipital cortex in DLB than AD. DLB was distinguished from AD more effectively on the basis of striatal distribution volume than occipital K1. DLB was distinguished from PD more effectively on the basis of cerebral cortical K1 than striatal distribution patterns

*DLB* Dementia Lewy bodies, *AD* Alzheimer’s disease, *PD* Parkinson’s disease, *FTD* frontotemporal dementia, *MCI* mild cognitive impairment, *HC* healthy controls, *PDD* Parkinson’s disease dementia, *MMSE* mini-mental state examination, *CI* cognitive impairment, *FTLD* frontotemporal lobar degeneration, *VaD* vascular dementia, *MSA* multiple system atrophy, *DLBD* diffuse Lewy body disease, *CDR* clinical dementia rating, *VMAT2* vesicular monoamine transporter type 2, *NC* elderly controls

### Metabolic imaging

[^18^F]FDG PET is used in detecting cerebral glucose metabolism, which is impaired in cases of neuronal degeneration and synaptic pathology. It has been widely used in assessing dementias, and has been proven to be an effective tool in aiding the diagnosis of AD and monitoring its progression [64–66].

In DLB, the topographical pattern of hypometabolism includes mainly the occipital areas, visual association cortices and the posterior parietotemporal areas [67–69], though in AD, decreased cerebral metabolism tends to involve other areas as well [70]. In a recent multimodal PET study assessing amyloid-β deposition and cerebral glucose metabolism, with [^11^C]PiB and [^18^F]FDG, respectively, Chinese patients with probable DLB exhibited cortical amyloid-β deposition, as well as hypometabolism in the temporo-parieto-occipital region, insular, precuneus, frontal lobe, posterior cingulate and caudate nuclei [71].

Another characteristic feature of DLB is preserved metabolism in the posterior cingulate area when compared to the precuneus and cuneus [72]. This is called the cingulate island sign and can be related to the common visual hallucinations in patients with DLB. Furthermore, it harbors a notable sensitivity and specificity [66, 73]. The cingulate island sign has been inversely correlated with neurofibrillary tangle pathology in autopsy studies [73]. A recent study has also reported association of cingulate island sign, not only with medial temporal lobe atrophy, but with clinical symptoms (cognitive impairment, visual hallucinations) of DLB patients as well [73].

### Imaging dopaminergic dysfunction

Dopamine transporter (DAT) imaging with SPECT using as a radiotracer [^123^I]FP-CIT-SPECT has been a valuable tool in assessing dopaminergic function in vivo. Decreased DAT uptake in basal ganglia is considered a supportive diagnostic feature according to current consensus diagnostic criteria [74, 75]. The diagnostic accuracy is even higher when applied in autopsy-proven cases of DLB [76–78]. Yielding a sensitivity of 88% and a specificity of 100% over non-DLB cases, [^123^I]FP-CIT-SPECT is a highly useful diagnostic tool [74]. A meta-analysis referring to 419 patients enrolled in 4 studies, showed a remarkable diagnostic accuracy, with a mean sensitivity of 86.5% and a mean specificity of 93.6% [79]. When comparing pathologically proven cases to clinical diagnosis, [^123^I]FP-CIT-SPECT has demonstrated increased accuracy in differentiating DLB from AD [80, 81]. In DLB, there is a decreased level of DAT, which is helpful in differentiating from AD where DAT is preserved [82, 83]. On the other hand, DAT imaging is not useful in discriminating DLB from PD-MCI and PDD, where there is a profound loss of DAT in the striatum [84]. Although DAT imaging possesses an inability to distinguish between parkinsonian syndromes, a recent study by Takaya et al. revealed that a combination of disease-specific perfusion patterns and striatal DAT activity accurately differentiates between atypical parkinsonian syndromes and Lewy body dementia [85]. However, in the rare cases of DLB where nigrostriatal degeneration is minimal and cortical pathology is the prominent feature, false negative results might occur. As for discriminating DLB patients from frontotemporal dementia or atypical parkinsonian syndromes (i.e. progressive supranuclear palsy (PSP), corticobasal degeneration (CBD)), [^123^I]FP-CIT-SPECT should not solely be accounted for as a reliable method of investigation [86]. The clinical phenotype should always be considered when interpreting findings regarding the above-mentioned conditions.

### Imaging cardiac sympathetic innervation

[^123^I]MIBG cardiac scintigraphy is widely used to assess cardiac postganglionic sympathetic degeneration, which is a common feature in neurodegenerative diseases with Lewy Bodies pathology. [^123^I]MIBG is a promising biomarker with the ability of excluding AD and predicting conversion of possible to probable DLB [87–89]. A large multicenter study including 133 patients, diagnosed according to the consensus criteria, highlighted similar sensitivity and specificity to [^123^I]FP-CIT-SPECT [90, 91]. Although the [^123^I]MIBG is a credible modality, certain pitfalls should be considered. The presence of diabetes mellitus or cardiac disease might provide false positive results [92]. Thus, such patients should be excluded from undergoing cardiac scintigraphy for diagnostic purposes. When comorbidities are taken into account, [^123^I]FP-CIT-SPECT may have a distinguishable diagnostic significance in the clinical setting [93].

### Amyloid imaging

Positive amyloid imaging is a classic feature of AD, with plaque deposition becoming apparent years after clinical symptomatology. Incorporation of amyloid imaging in AD consensus diagnostic criteria highlight the importance of such findings [94]. Furthermore, it may be proven elusive in early detection of disease pathology, disease monitoring and as a biomarker in disease-modifying trials with treatment targeting amyloid deposition. In DLB apart from α-synuclein aggregation, in some cases, pathology is also characterized by amyloid-β and tau deposition [95, 96]. The concurrence of the above-mentioned events leads to greater cognitive impairment [97].

Subsequently, imaging amyloid-β and tau deposition could potentially elucidate the association between AD-related pathology and α-synuclein aggregation. [^11^C]-Pittsburgh compound B ([^11^C]PiB) has been the most used radioligand to assess amyloid-β deposition in patients with DLB. Patients with DLB have shown increased [^11^C]PiB retention when compared to patients with PD or PDD and reduced retention when compared to patients with AD [67]. However, although the load of amyloid-β deposition cannot distinguish DLB from AD, it can be associated with the pace of cognitive decline in DLB patients [98, 99]. Other studies have associated amyloid pathology to the time-onset of cognitive features when related to parkinsonism [62]. Meta-analyses highlighted that 68% of patients with a diagnosis of probable DLB harbor abnormal [^11^C]PiB retention [67, 100]. Regarding differences between DLB and PDD, it has been demonstrated that cortical amyloid-β burden is significantly high in DLB patients, which is comparable to amyloid-β retention in AD, but conversely to PDD patients where amyloid-β pathology is scarce [62]. Dementia severity has been to shown to be trivial in the differential load of amyloid-β between DLB and PDD, with amyloid deposition possessing the ability to differentiate DLB and PDD, despite their overwhelming overlap in clinical, neuropathologic and neuropsychologic features [94]. Gomperts et al., through measuring [^11^C]PiB retention in Lewy body diseases such as DLB, PDD, PD and PD-MCI, found that amyloid-β burden was higher in DLB subjects compared to the other groups, with amyloid deposits being associated to cognitive impairment exclusively in DLB [97]. The early amyloid burden in DLB, comparative to PDD, may account for the variability in onset of dementia and parkinsonism between the two conditions. However, it is important to note that [^11^C]PiB binds to amyloid fibrils, but not soluble amyloid oligomers, thus the possibility remains that both DLB and PDD have high levels of toxic amyloid oligomers, which could potentially underlie cognitive impairment in both conditions. Notably, [^11^C]PiB retention patients with probable DLB or PDD, tend to have a similar pattern of cortical atrophy in MRI to patients with AD [101]. A recent study comparing [^11^C]PiB binding to GM atrophy rates concluded that higher retention at baseline was correlated to increase loss of GM, greater ventricular expansion and cognitive impairment [101]. In concordance with novel therapeutic strategies in AD, where amyloid pathology is targeted, amyloid imaging will have an upgraded role when anti-amyloid treatments are available for DLB patients as well.

### Tau imaging

The in vivo evaluation of tau pathology in DLB has been lacking until recently. The radioligand fluorine 18-labeled AV-1451 ([^18^F]AV-1451), also known as [^18^F]T807, has been proven suitable to assess tau deposition. Pathological studies have confirmed the predisposition of [^18^F]T807 for tau protein in neurofibrillary tangles instead of amyloid-β plaques or α-synuclein in Lewy bodies [102]. A recent study highlighted that cortical [^18^F]AV-1451 uptake was highly variable and greater than in the controls, especially in the inferior temporal gyrus and precuneus [102]. Furthermore, increased binding in these regions was found to be associated with cognitive impairment, as measured by the mini-mental state examination (MMSE) and the Clinical Dementia Rating scale [102]. These finding indicate a role for tau pathology in DLB pathogenesis. A subsequent larger [^18^F]AV-1451 PET imaging study reported that [^18^F]AV-1451 uptake was substantively more extensive and severe in AD compared to DLB patients [103]. [^18^F]AV-1451 uptake within the medial temporal lobe completely discriminated AD dementia from probable DLB, with AD exhibiting highest medial temporal uptake and DLB exhibiting the lowest. Probable DLB subjects had higher [^18^F]AV-1451 uptake in the posterior temporoparietal and occipital cortex compared to healthy controls, though no correlations were found between uptake in these regions and clinical measures such as motor parkinsonism, visual hallucinations, cognition or the presence of REM sleep behavior disorder (RBD). Global cortical [^11^C]PiB uptake, a marker of amyloid-β, was associated with elevated posterior temporoparietal and occipital [^18^F]AV-1451 uptake, indicating an atypical pattern of tau deposition in probable DLB [103]. Generally, there appears to be a gradient of increasing tau binding: from absent to minimal tau binding in cognitively normal PD, to low tau binding in PD patients with cognitive impairment, to intermediate tau binding in DLB and very high tau binding in AD [102, 104].

### Alpha-synuclein imaging

Pathological SNCA is detected in various forms, such as fibrils, Lewy bodies and oligomers. Moreover, SNCA deposits are abundant in other misfolded proteins, including tau and amyloid [105]. Thus, a radiotracer with high selectivity for SNCA over tau and amyloid is required to provide adequate accuracy. Other key features of a potential radiotracer include high affinity for SNCA aggregates, high penetration in the brain and prompt clearance.

Several potential compounds with a desirable profile and acceptable characteristics have been identified and the production of an accurate radiotracer for SNCA remains the greatest challenge of the neuroimaging community in movement disorders [106]. One of the first compounds that was tested in vitro was the benzoxazole BF227 [107]. Although [^18^F]BF227 harbored high affinity for amyloid and low affinity for SNCA in brain tissues, it was also evaluated in vivo in a cohort of MSA patients without fully overcoming interpretation issues [108]. A group of phenothiazine derivatives has also been investigated in animal studies, as potential compounds, due to their moderate selectivity for SNCA in PD brains [109]. [^18^F]WC-58a harbored a promising selectivity and affinity for synthetic SNCA fibrils; however, it proved to be too lipophilic with a slow clearance [110].

The development of a reliable SNCA radioligand is an unmet need regarding in-depth cohort stratification, monitoring disease progression and designing experimental treatments for synucleinopathies. The presence of incidental Lewy body disease among elderly is a caveat regarding the diagnostic utility of SNCA-PET. However, the capability of in vivo quantification could provide a valuable tool, especially when combined with other modalities to understand the full spectrum and progression of overlapping proteinopathies.

## Sensitivity and specificity of molecular imaging modalities in pathologically proven DLB cases

### [^123^I]-FP-CIT-SPECT

The importance of [^123^I]FP-CIT-SPECT in the differential diagnosis of DLB and non-Lewy body dementias has been extensively elucidated and is appreciated in the clinical setting [111].

Class I evidence have been provided regarding the application of [^123^I]FP-CIT-SPECT in discriminating DLB patients [112]. However, results should be replicated with patients recruited from different clinical settings. Reduced uptake yields a respectable diagnostic accuracy in discriminating DLB from AD. Alas, regarding differential diagnosis with atypical parkinsonian syndromes and frontotemporal dementia, the utility of [^123^I]FP-CIT-SPECT is limited [86, 113, 114].

There are scarce studies evaluating [^123^I]FP-CIT-SPECT alongside post-mortem tissue in DLB (Table 4). Among all cohorts, [^123^I]FP-CIT-SPECT exhibits higher sensitivity and specificity when compared to clinical diagnosis. Vascular lesions in the substantia nigra have been reported as a cause of false positive results [115]. Although positive scans have been reported in PSP, FTLD and CBD, diagnosis can typically be made on distinct clinical characteristics. However, Thomas et al. have reported two false positive cases with features of parkinsonism and a clinical diagnosis of DLB; post-mortem diagnosis revealed either AD or FTLD features without evidence of SNCA pathology in the substantia nigra [112]. The authors also identified six cases of false negative scans; three of the cases had a clinical diagnosis of AD at baseline without any signs of parkinsonism. At post-mortem examination, they harbored a mixed picture of AD and DLB features. The other three cases were retrospectively reassessed and actually fulfilled criteria for probable DLB. Hence, although [^123^I]FP-CIT-SPECT harbors a suitable accuracy, absence of an abnormal scan cannot fully exclude the presence of DLB. This discrepancy could be explained either by the fact that [^123^I]FP-CIT-SPECT measures the effect of SNCA in neurons and not the deposition of SNCA per se; thus cortical and striatal pathology might be evident without substantial nigrostriatal neuronal degeneration.

**Table 4:** Imaging modalities in pathologically proven DLB cases

Study	Imaging modality	Groups (based on clinical diagnosis)	Population	Interval between imaging and death (years)	Sensitivity (imaging vs postmortem)	Specificity (imaging vs postmortem)	Sensitivity (clinical diagnosis vs postmortem)	Specificity (clinical diagnosis vs postmortem)	Sensitivity (DLB vs AD)	Specificity (DLB vs AD)	Comments
Higuchi et al. [119]	[^18^F]FDG	7 DLB 11 AD, 10 NC	Miyagi Parkinson’s Disease Patient Registry	NA	NA	NA	NA	NA	86%	91%	A metabolic ratio of 0.92 in the visual association cortex was applied as a cutoff value (mean-2 SD) to discriminate DLB from AD
Minoshima et al. [120]	[^18^F]FDG	11 DLB (7 LBVAD, DLBD) 10 autopsy-confirmed-AD, 53 clinically diagnosed probable AD	Michigan Alzheimer’s Disease Re-search Center	3.4 ± 2.6	NA	NA	NA	NA	90%	80%	Only DLB patients harbored significant metabolic reductions in the occipital cortex, specifically in the primary visual cortex
Walker et al. [76]	[^123^I]FP-CIT-SPECT	13 DLB, 6 AD, 1 CBD, 16 HC	West-Essex Cohort	2.8 ± 1.8	88%	100%	75%	42%	NA	NA	The authors explored whether [^123^I]FP-CIT imaging improved the accuracy in discriminating dementia with Lewy bodies from non-dementia with Lewy bodies comparing clinical criteria with autopsy findings
Burton et al. [16]	Structural MRI	23 DLB, 11 AD, 12 VCI	Institute for Ageing and Health, Newcastle University	NA	NA	NA	NA	NA	91%	94%	The medial temporal lobe atrophy was found to serve as a diagnostic marker with high accuracy in differentiating AD from DLB and VCI
Lim et al. [66]	[^18^F]FDG	14 DLB (4 autopsy-confirmed), 10 AD (1 autopsy-confirmed)	Austin Health Memory Disorders and Neurobehavioural Disorder Clinics	NA	NA	NA	NA	NA	62% − 86%	100%	The cingulate island sign had the highest specificity, as opposed to hypometabolism in the lateral occipital cortex (88%)
Colloby et al. (2012)	[^123^I]FP-CIT-SPECT	7 DLB 12 PDD 4 AD	Community-dwelling referred to Old Age Psychiatry services	3.5 ± 1.7	NA	NA	NA	NA	NA	NA	It was shown that nigral dopaminergic cell loss (and not α-synuclein, tau or amyloid-β pathology) was associated with lower striatal [^123^I]FP-CIT uptake
Harper et al. [61]	Structural MRI	28 DLB, 101 AD, 55 FTLD, 55 HC	Queen Square Brain Bank, London; King’s College Hospital, London; VU Medical Centre, Amsterdam; Institute for Ageing and Health, Newcastle	NA	NA	NA	NA	NA	64%	82%	SVC classification accuracy demonstrated to be equivalent to or better than expert diagnosis, using six visual rating scales: medial temporal, posterior, anterior temporal, orbito-frontal, anterior cingulate and fronto-insula)
Thomas et al. [112]	[^123^I]FP-CIT-SPECT	33 DLB 22 AD	Newcastle Brain Tissue Resource Cohort	3.3 ± 2.3	80%	92%	87%	72%	NA	NA	Among patients with DLB, 10% (3 patients) met pathologic criteria for Lewy body disease but had normal [^123^I]FP-CIT imaging. While an abnormal [^123^I]FP-CIT scan supported Lewy body disease, an unremarkable scan did not exclude diagnosis especially when the brainstem was minimally involved. This study provided Class I evidence that [^123^I]FP-CIT scan accurately identifies patients with DLB

*LBVAD* Lewy body variant of AD, *DLBD* pure diffuse Lewy body disease, *VCI* vascular cognitive impairment, *FTLD* frontotemporal lobar degeneration, *SVC* Support vector classifier, *SD* standard deviation. exclude diagnosis

### Amyloid imaging

A study by Albin et al., combining amyloid and dopamine terminal PET imaging, revealed that imaging classifications were concordant with neuropathological diagnostic classifications in 33/36 cases (91.7%) [116]. Of three cases with discordant imaging-pathological classification, one had a clinical and imaging diagnosis of DLB, but a pathological diagnosis of AD. However, alpha-synuclein immunoreactive Lewy body inclusions were present in the midbrain, thus suggestive of mixed AD-DLB pathology. The other discordant subject was classified as DLB via imaging, but within the frontal cortex and hippocampus, had transactive response DNA binding protein 43 kDa (TDP-43)-immunoreactive neurites. This was particularly unusual given that this case exhibited unilateral striatal loss of [^11^C]DTBZ, a marker of striatal dopamine terminal integrity. Although 8.3% of cases differed in diagnostic classifications based on neuroimaging and histopathology, this was an improvement compared to their previous studies, which demonstrated that ~ 35% of cases had discordant expert clinical consensus and imaging classifications [117, 118]. Therefore, this combined imaging approach may be useful in establishing more accurate markers for differentiating dementias.

### [^18^F]FDG PET

Patterns of cerebral glucose metabolism in DLB have been reported to encompass the ability to differentiate DLB from other forms of dementia. Although DLB has shown to exhibit widespread glucose hypometabolism across cortical regions, metabolic reduction has been shown to be most prominent within the visual association cortex. Through looking at metabolism within this region, DLB can be distinguished from AD with a sensitivity of 86% and specificity of 91% [119]. Although these authors only scanned one patient with autopsy-confirmed DLB diagnosis, postmortem results from 17 DLB brains revealed a distinct and extensive white matter spongiform change with coexisting gliosis throughout cerebral white matter. These changes were consistently and pronouncedly observed within the occipital lobe, with the severity of the regional spongiform change mainly corresponding to the regional differences in patterns of reduced glucose metabolism illustrated by living AD and DLB patients [119]. This study is in corroboration with findings reported by Minoshima et al., who revealed that autopsy-confirmed AD and DLB patients exhibited regional metabolic reductions, specifically within posterior cingulate, parietotemporal association, and frontal association cortex. DLB cases, in particular, demonstrated significant metabolic reductions within the occipital cortex, specifically within the primary visual cortex, which had the ability to distinguish DLB from AD with a specificity of 80% and sensitivity of 90% [120]. Furthermore, patients who were initially clinically diagnosed with probable AD, but later fulfilled the clinical criteria for DLB, demonstrated hypometabolism within the primary visual cortex at higher frequencies, which often preceded the manifestation of several DLB symptoms [120]. Although the authors of these studies argue that [^18^F]FDG PET may be a useful tool to distinguish DLB from other dementias, Albin et al. demonstrated that ~ 30% of classifications, based on glucose metabolism, differed from final neuropathological diagnoses in a cohort of DLB, AD and FTD who underwent PET imaging and subsequent autopsy [116]. They reported 2 cases where [^18^F]FDG PET classification was AD but pathological verification was DLB, though combined amyloid and dopaminergic terminal PET imaging correctly identified the pathological diagnosis [116]. Therefore, the authors argued that classifications based on [^18^F]FDG PET are less precise, with misclassifications ascribed to [^18^F]FDG PET being due to the absence of occipital metabolic deficits in a substantial proportion of DLB patients [121].

A proposed [^18^F]FDG PET imaging feature of DLB is the cingulate island sign, which refers to the sparing of the posterior cingulate relative to the precuneus and cuneus. This sign is said to be useful for an accurate diagnosis of DLB, given that it is specific, with a reasonable sensitivity [66]. Studies assessing this sign in clinically diagnosed DLB have revealed that the cingulate island sign metabolism, as measured by [^18^F]FDG PET, is highly specific for detecting DLB, with a specificity of 100% and sensitivity ranging from 62 to 86% [66]. In this study, 4/14 subjects had autopsy-confirmed DLB, with the others being followed clinically for several years and their diagnosis remaining unchanged. Similarly, the cingulate island sign metabolism is higher in DLB patients compared to AD, independent of amyloid load [72]. Patients who exhibited the cingulate island sign were more likely to be classified as having high or intermediate probability of DLB pathology, receiving a clinical diagnosis of DLB. Furthermore, a higher cingulate island sign ratio was associated with a lower burden of neurofibrillary tangles. 2 subjects who had the lowest cingulate island sign ratio where clinically diagnosed with DLB, but at autopsy, exhibited high likelihood of AD pathology without Lewy body pathology. Taken together, these results indicate that a reduction in cingulate island sign ratio is associated with high burden of AD-type neurofibrillary tangles, therefore ‘pure’ DLB would present with the typical cingulate island sign. This is incredibly important, as the convergence and co-occurrence of AD and DLB pathology is common, with ‘pure’ DLB accounting for no more than a third of all DLB cases and possibly 10% of all clinical dementia cases. This was demonstrated by Barker et al., who identified DLB in 14–26% of dementia cases, with the ‘pure’ form of DLB accounting for 0–19% of dementia patients [122]. Therefore, whilst helpful, the cingulate island sign will not very sensitive or specific for DLB in other pathological series.

## Conclusions

DLB is a common dementia in older patients and differential diagnosis with AD and especially PDD can be challenging. Well-established neuroimaging modalities such as [^123^I]FP-CIT-SPECT and [^123^I]MIBG can be extremely useful in adding diagnostic accuracy between DLB and AD but not with PD-MCI and PDD or atypical parkinsonian syndromes. The application of novel radioligands targeting pathways relevant to underlying pathophysiology, can provide valuable tools in exploring molecular pathology. Furthermore, precise quantification of tau pathology and the possibility of a tracer targeting α-synuclein will further expand insights and potentially harbor innovative therapeutic opportunities.
